# Deferasirox’s Anti-Chemoresistance and Anti-Metastatic Effect on Non-Small Cell Lung Carcinoma

**DOI:** 10.3390/biomedicines12102272

**Published:** 2024-10-07

**Authors:** Yamixa Delgado, Anamaris Torres-Sanchez, Daraishka Perez, Grace Torres, Sthephanie Estrada, Natalia Ortiz Alvelo, Jaisy Vega, Laurie Santos, Aracelis Torres, Bismark A. Madera, Yancy Ferrer-Acosta

**Affiliations:** 1 Biochemistry & Pharmacology Department, San Juan Bautista School of Medicine, Caguas, PR 00725, USA; daraishkapc@sanjuanbautista.edu (D.P.); gracetr@sanjuanbautista.edu (G.T.); nataliaoa@sanjuanbautista.edu (N.O.A.); jaisyvo@sanjuanbautista.edu (J.V.); aracelistr@sanjuanbautista.edu (A.T.); 2 Biology Department, University of Puerto Rico-Rio Piedras, San Juan, PR 00925, USA; anamaris.torres@upr.edu (A.T.-S.); sthephanie.estrada@upr.edu (S.E.); 3 Biomedical Graduate Program, Universidad Central del Caribe, Bayamón, PR 00960, USA; 424lsantos@uccaribe.edu; 4 Molecular Sciences Research Center, University of Puerto Rico, San Juan, PR 00926, USA; bismark.madera@upr.edu; 5 Department of Anatomy and Neurobiology, School of Medicine, University of Puerto Rico, Medical Sciences Campus, San Juan, PR 00936, USA; yancy.ferrer@upr.edu

**Keywords:** deferasirox, iron chelators, ribonucleotide reductase, metastasis, chemoresistance, non-small cell lung carcinoma

## Abstract

Clinically approved iron chelators, originally designed to address iron overload disorders, have emerged as potential anticancer agents. Deferasirox (Def), a tridentate iron chelator, has demonstrated antiproliferative effects in cancer. **Background/Objectives**: This study aims to elucidate the mechanism of action of Def and its impact on non-small cell lung carcinoma (NSCLC). **Methods**: NSCLC A549 cells were treated with Def to assess cytotoxicity, the effect on nuclear and mitochondrial pathways, and iron-containing proteins and genes to evaluate anti-metastasis and chemoresistance. A lung carcinoma mouse model was used for in vivo studies. **Results**: Our findings revealed that Def induced cytotoxicity, effectively chelated intracellular iron, and triggered apoptosis through the increase in phosphatidylserine externalization and caspase 3 activity. Additionally, Def caused G0/G1 cell cycle arrest by downregulating the ribonucleotide reductase catalytic subunit. Furthermore, Def perturbed mitochondrial function by promoting the production of reactive oxygen species and the inhibition of glutathione as a measurement of ferroptosis activation. Def demonstrated inhibitory effects on cell migration in scratch assays, which was supported by the upregulation of *n-myc downstream-regulated gene 1* and downregulation of the epidermal growth factor receptor protein. Also, Def downregulated one of the main markers of chemoresistance, the *ABCB1* gene. In vivo experiments using a lung carcinoma mouse model showed that Def treatment did not affect the animal’s body weight and showed a significant decrease in tumor growth. **Conclusions**: This investigation lays the groundwork for unraveling Def action’s molecular targets and mechanisms in lung carcinoma, particularly within iron-related pathways, pointing out its anti-metastasis and anti-chemoresistance effect.

## 1. Introduction

Iron is an essential trace element for cellular development and proliferation. This element has an important role in several cellular processes in the nucleus (e.g., DNA synthesis, DNA replication, and nucleic acid repair), in the mitochondrion (e.g., electron transport chain (ETC) and ATP production), and in the synthesis of heme and iron–sulfur clusters [1,2]. Thus, iron homeostasis is finely regulated at cellular and systemic levels. Several studies found an association between the dysregulation of iron homeostasis and different types of cancer, such as breast, colorectal, lung, pancreatic, and prostate [1,2,3,4]. Thus, cancerous cells have a higher requirement of iron, inducing an enhanced uptake of iron than in normal cells to sustain the aberrant overgrowth and proliferation [4].

Cancer is a major public health issue, ranking as a leading cause of death worldwide [5]. For the past decades, lung cancer has been the most diagnosed cancer (~237,000 new cases in 2022) and the leading cause of cancer death in the USA [6,7]. Non-small cell lung cancer (NSCLC) is the most common epithelial lung cancer over small cell lung cancer (SCLC), accounting for about 85% of all lung cancer types [8]. Thus, the development of advanced early detection techniques and novel therapeutic approaches remains essential. Among the existing clinical drugs, several are currently under investigation for repurposing in cancer treatment.

**Figure 1:**
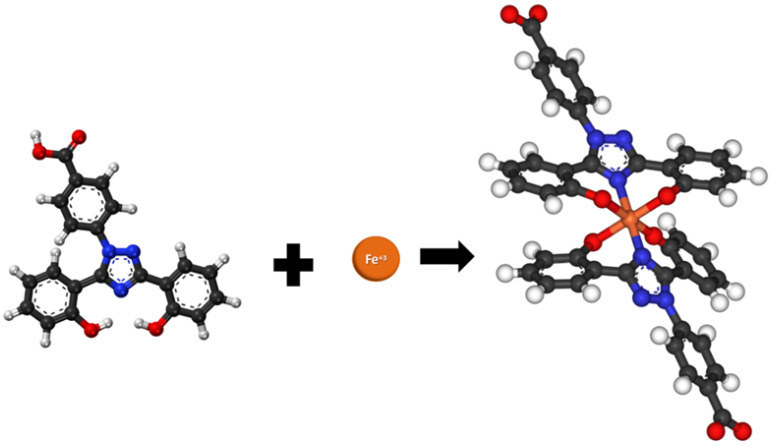
Iron chelation by Def. Two molecules of Def bind an iron (Fe^+3^) atom to form an octahedral structure. (Ball and stick model color code: terracotta-iron, red-oxygen, dark gray-carbon, blue-nitrogen, white-hydrogen). Adapted from Wikipedia and available at https://en.wikipedia.org/wiki/Deferasirox# (accessed on 8 March 2024). All structured data images from Wikipedia are available under the Creative Commons CC0 License (No copyright).

Iron chelators have been used extensively to treat iron overload disorders to help patients evade the effects of iron toxicity [9]. Thus, repurposing iron chelators targeting iron metabolism in cancer emerges as a novel therapeutic approach. Studying several iron chelators, it was proposed that they selectively deplete cancer cells of iron based on the increased iron concentrations in these cells [10,11]. Despite the creation of many synthetic iron chelators, only three are commonly used in the clinical setting: deferoxamine, deferasirox (Def), and deferiprone because of their high effectivity [12]. Def (commercially known as Exjade^®^) is a tridentate iron chelator that was found to exhibit anti-proliferative effects on various cancer cell lines [13]. Molecularly, Def has a hydroxyphenyl-triazole that needs two molecules to form a stable complex with a single iron ion (Figure 1) [12,13]. Although Def has demonstrated side effects, the incidence of serious adverse effects appears to be low and is generally well tolerated in adults and children [12]. In previous studies, Def was shown to inhibit the ETC, leading to reactive oxygen species (ROS) production in ovarian cancer [14]. In this way, Def could disrupt the functionality of iron-containing proteins in the nucleus and mitochondria [15]. Several of the iron-regulated proteins are ribonucleotide reductase (RR), key for DNA synthesis [16]; ETC complexes, key for mitochondrial membrane potential [17]; and the N-myc downstream-regulated gene 1 (NDRG1), known as the metastasis suppressor [18], which can be affected by Def.

RR is the enzyme that catalyzes the conversion of ribonucleotides to deoxyribonucleotides, which is the rate-limiting step for DNA synthesis [16]. Thus, RR is vital for the maintenance of a balanced nucleotide pool intracellularly, making it a key target of interest in cancer [19]. RR consists of two subunits: a catalytic α subunit (RRM1) and a radical-generating β subunit (RRM2) which contains a diiron cofactor [20]. Both subunits are known to increase in a variety of cancers including NSCLC [19,20].

The *ABCB1* gene (translated as the P-glycoprotein), part of the ATP-binding cassette transporter family, has been correlated with poor prognosis and cancer recurrence in lung cancer [21]. This protein is also known as the multidrug resistance protein or efflux pump protein. It was reported that when *ABCB1* is overexpressed, it is associated with cancer-stem cell-like properties and with the epithelial–mesenchymal transition [22]. Moreover, researchers demonstrated that *ABCB1* downregulation can help overcome drug resistance in NSCLC [23].

NDRG1 has been shown to potently suppress metastasis in a variety of cancer types [24]. It was shown that NDRG1 is widely expressed in normal cells while downregulated in many types of cancer [25]. NDRG1 promotes the formation of adherent junction proteins such as E-cadherin and B-catenin, resulting in reduced metastatic potential [26]. The expression at the mRNA and protein levels of NDRG1 could be downregulated by the effect of iron chelators in cancer, highlighting the regulatory role of intracellular iron [26]. Thus, NDRG1 was implicated as the primary target of iron chelators [10].

The epidermal growth factor receptor (EGFR) is critical in NSCLC due to its central role in driving tumor progression and proliferation [22]. EGFR overexpression is often associated with enhanced metastatic potential and resistance to chemotherapy, as the receptor’s signaling pathways promote survival under therapeutic stress [27]. Thus, inhibiting the expression of EGFR and tailoring targeted therapies that can inhibit this oncogenic process is currently a key target in lung cancer.

Although Def is currently studied for different types of cancer [28,29], Def’s molecular targets and mechanisms of action remain to be completely elucidated, especially in NSCLC. Here, we studied Def against NSCLC, focusing particularly on the mechanisms that target iron-containing and iron-regulated proteins in the nucleus and mitochondria. Our study shows that Def induced cellular iron-depletion, the downregulation of RR and ABCB1 genes, the downregulation of EGFR protein, G0/G1 cell cycle arrest, the disruption of mitochondrial membrane, an increase in reactive oxygen species (ROS), the induction of ferroptosis, a decrease in cell migration area, and the upregulation of the NDRG1 in A549 NSCLC cells. In vivo experiments confirmed the safety and effectiveness of Def, significantly decreasing tumor growth in a non-immunocompromised mouse model of NSCLC.

## 2. Materials and Methods

### 2.1. Reagents

High-quality sterile nanopure water (resistivity of 18.2 MΩ cm, 121 °C and 18 PSI) produced from a Thermo Scientific™ Barnstead™ Easypure™ II system (Thermo Fisher Scientific, Waltham, MA, USA) was used to prepare all aqueous solutions. Deferasirox (Exjade^®^, Def) was generously provided by Novartis^®^ (Basel, Switzerland) through a material transfer agreement. Essential cell culture components including Dulbecco’s Modified Eagle’s Medium (DMEM-high glucose, D6429), L-glutamine (1917006), fetal bovine serum (FBS; F0926), phosphate-buffered saline (PBS, D8537), MEM non-essential amino acid solution (M7145), ferric citrate (F3388), a 2′,7′-dichlorofluorescein diacetate (DCFHDA; 35845) probe, a TRI RNA Isolation Reagent^®^ (T9424), an Extracellular Matrix Growth Factor Reduced Gel (ECM-GFRG) from Engelbreth-Holm-Swarm murine sarcoma (E6909), DNase-/RNase-Free water (W4502), a Total Iron-Binding Capacity (TIBC) and Serum Iron Assay Kit (MAK394) and a penicillin/streptomycin/amphotericin antibiotic (A5955) solution were sourced from MilliporeSigma (St. Louis, MO, USA). Cell lines A549 (human lung carcinoma; ATCC CCL-185), NL20 (human normal bronchus; ATCC CRL-2503), LLC (Lewis lung carcinoma; ATCC CRL-1642), MRC5 (human fibroblast; ATCC CCL-171), Bronchial Epithelial Cell Growth Kit (epinephrine, transferrin, triiodothyronine, hydrocortisone, EGF, and insulin) (PCS-300-040) were obtained from the American Type Culture Collection (ATCC) (Manassas, VA, USA). The CellTiter 96 Aqueous Non-Radioactive Cell Proliferation Assay (G358C) and Apo-ONE^®^ Homogeneous Caspase-3/7 Assay (G7792) were obtained from the Promega Corporation (Madison, WI, USA). A GSH Assay Kit (ab239709) was bought from Abcam (Cambridge, UK). Kits for flow cytometry analysis (cell cycle (MCH100106), DNA damage (MCH200107), EGFR Activation Dual Detection Kit (MCH200102) and MitoPotential (MCH100110)) were purchased from Luminex Corporation (Austin, TX, USA). Fluorescent dyes such as Annexin V Alexa Fluor 488 Ready Flow Reagent (R37174) and NucBlue Live Cell Stain ReadyProbes (R37606) for confocal microscopy were acquired from Thermo Fisher Scientific^®^. High-Capacity cDNA Reverse Transcription Kit (4374966) and the following primers: *GAPDH* (Assay ID: Hs99999905_m1), *NDRG1* (Assay ID: Hs00906878_m1), ribonucleotide reductase *RRM1* (Assay ID: Hs01040697_m1), and *ABCB1* (Assay ID: Hs01070641_g1), for TaqMan gene expression assays were purchased from Applied Biosystems (Waltham, MA, USA) and Thermo Fisher Scientific^®^. All other chemicals used were of analytical grade and procured from various suppliers without additional purification.

### 2.2. Stocks and Dilutions

For in vitro experiments, Def was initially dissolved in dimethyl formamide (DMF, 227056) to create a stock solution. This stock solution was subsequently diluted in PBS, where the final DMF concentration in cells did not exceed 1%.

### 2.3. Cell Culture Conditions

Human NSCLC A549, normal bronchus NL20, and fibroblast-like MRC5 (cancer-associated fibroblasts (CAF)) cell lines were harvested mostly following ATCC guidelines (i.e., DMEM-high glucose, 1% L-glutamine, 10% FBS, and 1% antibiotics under 5% carbon dioxide (CO_2_) and 95% air at 37 °C). NL20 cells were cultured under the same conditions with additional supplements: 1% non-essential amino acids, 1.0 µM epinephrine, 5 µg/mL transferrin, 10 nM triiodothyronine, 0.1 µg/mL hydrocortisone, 5 ng/mL EGF, and 5 µg/mL insulin. We ensured the quality of our cultured cells through mycoplasma contamination detection, population doubling time, and morphological changes. Mycoplasma contamination was tested periodically for all our cell lines and was not detected. Cell morphology was observed under the microscope before every passage and compared with standard morphological characteristics for each cell line. STR profiles were provided by ATCC. Cells with less than 25 passages were used for our experiments. Untreated cells with 1% DMF served as negative controls, while positive controls included cells treated with 20% DMF or other specific compounds like peroxide (H_2_O_2_), depending on the experiment.

### 2.4. Cell Viability Assay

To determine the 50% inhibitory concentration (IC_50_) of Def in A549 NSCLC and MRC5 normal lung cells, we followed our previously published protocol [30]. Briefly, A549, NL20, and MRC5 cells were seeded into 96-well plates at a density of 1 × 10^5^ cells/mL in a supplemented DMEM medium. After 24 h, the cells were treated with various concentrations of Def (10, 25, 50, 75, 100, 300 μM) and incubated for another 24 h. Subsequently, a volume of 10 μL of the MTS reagent from the CellTiter 96^®^ kit was added to each well, followed by a 1 h incubation at 37 °C in a 5% CO_2_ atmosphere. Absorbance was then measured at 492 nm (n = 8) using a Thermo Scientific Multiskan FC microplate reader spectrophotometer. This assay was conducted in three independent experiments.

Cell viability was calculated using the formula:

% Viability = (Def treated cells Abs − Def incubation media Abs)/(untreated cells Abs − incubation media Abs) × 100

IC_50_ values were derived from dose–response curves of the mean ± SD, with data normalized using a non-linear fit of log (drug inhibition) vs. normalized response-variable slope to obtain the best-fit IC_50_ and R-squared values.

### 2.5. Confocal Microscopy

For this experiment, we followed our previously published protocol [31]. A549 cells were seeded in cover slip plates at a density of 1 × 10^4^ cells/mL in supplemented DMEM and incubated for 24 h. Subsequently, the cells were treated with Def (at IC_25_) for another 24 h. After treatment, the cells were washed with PBS and fixed with a 3.7% formaldehyde solution for 15 min. Following fixation, the cells were washed twice with PBS and then incubated with NucBlue nuclear stain and Annexin V Alexa Fluor 488 solutions at room temperature for 20 min. The samples were then analyzed using a Nikon Eclipse Ti confocal microscope (Nikon Instruments Inc., El Segundo, CA, USA) equipped with a 40× oil objective with a numerical aperture of 1.3. The microscope used a filter set of Ex/Em = 405/460 nm for NucBlue DAPI and Ex/Em = 488/525 nm for Annexin V Alexa Fluor, while a photomultiplier tube collected emission light. The fluorescence intensity was quantified using the NIS-Elements Viewer program (version 5.21 64-bit (Nikon Healthcare Business Division, Tokyo, Japan) using an automated measurement module. The fluorescence intensity of untreated cells (negative control) was used for the calibration of each fluorescence channel (green and blue). This calibrated intensity was then used to analyze all treated cells. This assay was conducted in duplicate across two independent experiments.

### 2.6. Iron Binding Assay

To measure iron levels, we utilized the total iron-binding capacity (TIBC) and serum iron assay following the manufacturer’s instructions. A549 cells were seeded in 96-well plates at a density of 1 × 10^5^ cells/mL in growth medium supplemented with iron citrate (200 μM). After 24 h, the medium was changed, and Def (IC_50_) was incubated with the cells for 1 h. The cells were then washed and incubated for 10 min in the TIBC assay buffer. Following this incubation, the TIBC detector was added, and the cells were incubated for an additional 10 min. Absorbance was measured at 570 nm (n = 4) using a Thermo Scientific Multiskan FC microplate reader spectrophotometer. This assay was performed in quadruplicate across three independent experiments. The intracellular free iron was calculated using the following formula:

% Free iron = (Def treated cells Abs − Def incubation media Abs)/(untreated cells Abs − untreated incubation media Abs) × 100.

### 2.7. Total Glutathione (GSH) Assay

To assess the total GSH levels, a colorimetric assay was performed following the manufacturer’s protocol. A549 cells were seeded in 6-well plates at a density of 1 × 10^6^ cells/mL. After 24 h, cells were treated with Def (IC_50_) and with iron citrate (200 μM) as a positive control group. Cells were harvested by centrifugation for 5 min at 4 °C, washed with PBS, and lysed in buffer. Following lysis, a solution of 5% sulfosalicylic acid was added, and the samples were centrifuged for 10 min to obtain the supernatant. A GSH standard curve was generated ranging from 0 to 50 µg, diluted with reaction buffer and a concentration of 1% sulfosalicylic acid. The reaction mix of NADPH generating mix, glutathione reductase, and reaction buffer was prepared and added to a 96-well plate. After a 10 min incubation, the Def-treated, positive control, untreated samples and standards were added, followed by the substrate solution. The reaction was incubated for 5–10 min at room temperature, and absorbance was measured at 405 nm. All experiments were performed in quadruplicate in two independent experiments for accuracy. GSH concentrations were determined by interpolating the absorbance values against the standard curve and then converted to % GSH using the following formula:

% Total GSH= (Def treated cells Abs − Def incubation media Abs)/(untreated cells Abs − untreated incubation media Abs) × 100.

### 2.8. Total Oxidative Stress Assay

The production of total ROS was measured using the DCFHDA probe to yield the highly fluorescent 2′,7′-dichlorofluorescein (DCF). A549 cells were seeded at a density of 1 × 10^5^ cells/mL into black well plates with clear bottoms. After 24 h, cells were treated with Def (at IC_50_) and controls for another 24 h. Subsequently, we added 50 μL of the DCFH-DA working solution (10 μM) to each well and incubated them for 1 h at room temperature. The fluorescence of each well was then measured at Ex/Em = 485/530 nm using a Tecan^®^ M200 plate reader. This assay was performed in triplicate across three independent experiments. GraphPad Prism 9 (San Diego, CA, USA) was used to analyze the mean ± SD of three measurements of each condition.

### 2.9. Caspase 3 Activity Assay

Caspase 3/7 enzymatic activity was assessed as a measurement of intrinsic apoptosis using the Apo-ONE^®^ kit. A549 cells were seeded at a density of 1 × 10^5^ cells/mL into black 96-well plates with clear bottoms for 24 h. Then, cells were treated with Def (at IC50) and controls for another 24 h. Following the incubation period, a volume of 50 μL of (rhodamine 110-Z-DEVD-R110) substrate 1:100 in lysis buffer was added to each well. The plate was gently shaken at 150 rpm for 2 h at room temperature. Subsequently, the fluorescence at Ex/Em = 485/530 nm was measured using a Tecan^®^ M200 plate reader. This assay was performed in triplicate across three independent experiments. GraphPad Prism 9 was used to analyze the mean ± SD of three measurements of each condition.

### 2.10. Flow Cytometry

Flow cytometry was employed to investigate several cellular mechanistic pathways in A549 cells post-incubation with Def, following a similar published protocol [30]. Briefly, the cells were seeded with supplemented DMEM in a 6-well plate at a density of 1 × 10^6^ cells/mL for 24 h incubation, and the A549 cells were treated with Def using a concentration to ensure a clear signal without causing excessive cell death. Experiments were performed using a Muse^®^ Cell Analyzer (EMD Millipore Corporation, Temecula, CA, USA), calibrated with the Muse^®^ System Check Kit (Luminex MCH100101) before each use. Each experiment was performed at least twice, with samples analyzed in triplicate. The capillary line was washed after each run to prevent contamination, and the Muse^®^ Cell Dispersal Reagent (Luminex 4100-1790) was used to avoid cell doublets.

#### 2.10.1. Cell Cycle Arrest

A549 cells were treated with Def (IC_50_) for 24 h. Subsequently, the cells were scraped, centrifuged, and fixed. The cell pellet was then stained with a propidium iodide (PI)-based cell cycle reagent and incubated for 30 min for immediate measurement.

#### 2.10.2. DNA Damage

A549 cells were exposed to Def (IC_50_) for 24 h. Following treatment, the cells were scraped, centrifuged, and suspended in a fresh assay buffer, fixed and permeabilized, after which an antibody cocktail (anti-pH2AX and anti-pATM) was added and incubated for 30 min. The cells were then washed and suspended in a fresh assay buffer for measurement.

#### 2.10.3. Mitochondrial Membrane Potential

Mitochondrial permeabilization was assessed using the MitoPotential Kit. After incubating A549 cells with Def (IC_50_) for 24 h, the Mito-cationic lipophilic dye and the TM 7-AAD cell death marker were added and incubated for 30 min at 37 °C for immediate measurement.

#### 2.10.4. EGFR Protein Expression and Activation

A549 cells were exposed to Def (IC_50_) for 24 h. After Def incubation, the cells were scraped and centrifuged, and the pellet was resuspended in the assay buffer of the EGFR Kit. Then, the pellet of cells was fixed and permeabilized. After centrifugation, the antibody cocktail composed of anti-phospho-EGFR (Tyr1173)-Alexa Fluor^®^ 555 and an anti-EGFR-PECy5 was added to the cells for 30 min to measure the total levels of EGFR.

### 2.11. Scratch Wound Healing Migration Assay

The migratory capacity of A549 NSCLC cells was assessed following a 24 h incubation with Def (IC_10_). The cells were cultured as previously described [30], seeded in 6-well plates at a density of 2 × 10^6^ cells/mL, and incubated for 24 h in a DMEM growth medium until reaching 90% confluence. Subsequently, a scratch was created in the confluent cell layer using a sterilized 200 μL pipette tip. Detached cells were eliminated with two PBS washes. The cells were then treated with Def and further incubated for 24 h. The wound area was measured before and after treatment using NIH ImageJ program version 1.53e (Bethesda, MA, USA) to quantify cell migration, with comparisons made to the control group (untreated cells). This assay was conducted in duplicate across three independent experiments.

### 2.12. mRNA Gene Expression

RNA Extraction. NSCLC A549 cells were seeded at a density of 1 × 10^6^ cells/mL into 6-well plates. Cells were treated with Def (IC_25_) for 24 h. Total RNA was extracted from the cells using TRI Reagent^®^ (TRIzol) according to the manufacturer’s protocol. RNA concentration and purity were assessed using a NanoDrop spectrophotometer (Thermo Fisher Scientific). Subsequently, reverse transcription to synthetize the cDNA was performed using the Reverse Transcription Kit mixing 2 μg of RNA with random primers, deoxynucleotide triphosphates (dNTPs), and reverse transcriptase with the assay buffer in a thermal cycler.

Real-Time-quantitative PCR. An mRNA-based gene expression was conducted in a StepOnePlus^®^ Real-Time PCR System (Thermo Fisher Scientific) using *NDRG1*, *RRM1*, and *ABCB1* primers of the TaqMan™ Gene Expression Assay. Our PCR reaction mixture was prepared with 10 ng of cDNA, TaqMan™ Fast Advanced Master Mix (44-445-57) and the respective TaqMan primers. PCR cycling conditions consisted of an initial denaturation at 95 °C for 10 min, followed by 40 cycles of denaturation at 95 °C for 15 s and an annealing/extension at 60 °C for 60 s. Relative expression levels were calculated using the comparative Ct (ΔΔCt) relative quantitation, normalizing with glyceraldehyde-3-phosphate dehydrogenase (*GAPDH*) (housekeeping endogenous control). These assays were performed in triplicate across two independent experiments.

### 2.13. In Vivo Assays

Cell Growth and Implant of Lewis Lung Carcinoma (LLC) for Tumor Development in Mice. Following procedures by Barcelo-Bovea et al., 2020 and Dominguez et al., 2022 [32,33]), LLC cells were cultured using the same culture conditions as previously described. The cells were grown to confluency, then gently scraped off the plate, pelleted, and quantified.

Tumor Induction in Mice and Treatments. The mouse strain used in these experiments to develop the syngeneic mouse model was C57/BL6J (Jackson Labs, Bar Harbor, ME, USA), which was compatible with the mouse strain of the LLC cells. Adult male mice (32–60 weeks) compatible with the reproductive senescence [33] were selected because this is an age representing NSCLC patients’ stage of disease. The mice were implanted with LLC cells to facilitate the growth of a tumor over a period of 12 days. Mice were anesthetized with a solution of 2% isoflurane and subcutaneously injected with 400 μL of 1 × 10^7^ LLC cells prepared in a solution of 50% ECM-GFRG and 50% supplemented media. Cell injection was administered into the upper right dorsal area of each mouse. Mice were injected subcutaneously with Def at 20, 35, 45, and 90 mg/kg (n = 1 for each dose; total treated n = 4; vehicle n = 4) every 3 days after tumor implant up to day 12. The maximum tumor volume is limited to 10% of the animal’s body weight (~30 g mice/<2300 mm^3^ tumor volume) regulated by Institutional Animal Care and Use Committees (IACUC) and NIH’s Office of Laboratory Animal Welfare (OLAW). The euthanasia method for mice was through inhaled anesthesia (4%) and swift decapitation. This method of euthanasia is approved by the AVMA Guidelines and the NIH OLAW. All necessary approvals from the Universidad Central del Caribe Institutional Animal Care and Use Committee (IACUC) were in place for the performed research: Assurance ID number D16-00343; IACUC Protocol Universal Number 048-2019-13-01-PHA-IBC. Tumor volume was measured manually with a caliper on days 3, 6, 9, and 12 using the following formula:

Tumor volume (mm^3^) = length × (width)^2^/2

Tumor growth was calculated from day 3 and day 12 using the following formula:

% Tumor growth = (VolumeDay12 − VolumeDay3)/(VolumeDay12) × 100

### 2.14. Statistical Analyses

In vitro assays. The data are presented as mean ± SD of measurements for each treatment condition and were analyzed using GraphPad Prism 9 software (San Diego, CA, USA). For the caspase 3 and free iron assay, statistical comparisons between treated and control (untreated) cells were performed using Student’s *t*-test (Welch’s correction for unequal variances). For the ROS and GSH assay, statistical analysis was performed using a one-way ANOVA with Dunnet comparison. For flow cytometry assays comparing the activation of several cellular metabolic populations, statistical analysis was performed using a two-way ANOVA with Sidak comparison. Statistical significance (*p*-values) levels were defined as follows by the software: **** for *p* < 0.0001, *** for *p* ranging from 0.0001 to 0.001, ** for *p* between 0.01 and 0.001, * for *p* from 0.05 to 0.01, and ns (non-significant) for *p* > 0.05.

In vivo assays. A total of n = 4 mice were treated with vehicle (PBS), and n = 4 mice were treated with Def. Each mouse was treated with a specific Def concentration 20, 35, 45, and 90 mg/kg. A two-tailed, paired Student’s *t*-test between Vehicle-treated and Def-treated mice tumor growth percent was performed using GraphPad Prism 9 and showed statistical significance within a 95% confidence interval at *p* < 0.05.

## 3. Results

### 3.1. Def Induces Cell Death and Apoptosis in NSCLC Cells

**Figure 2:**
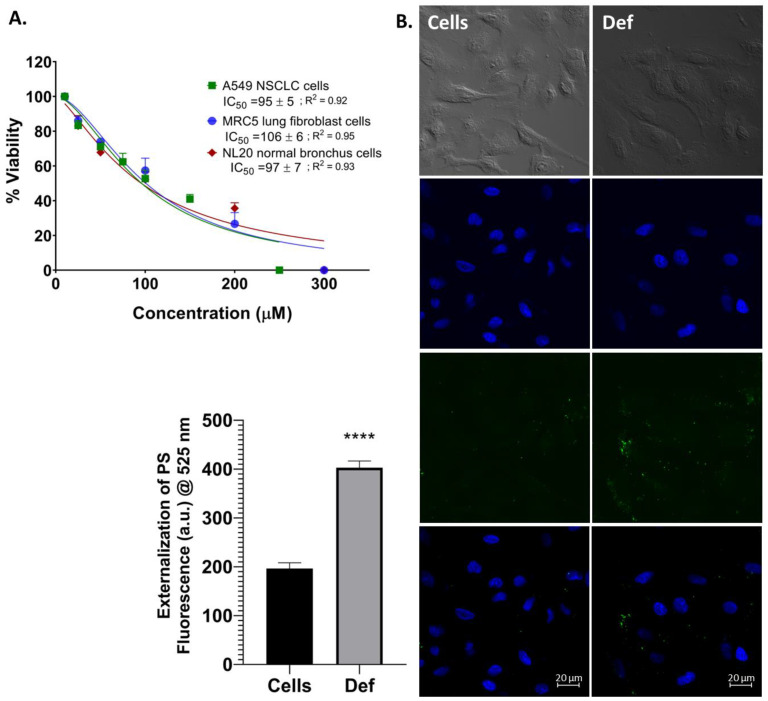
Def’s cytotoxicity. (**A**) MTS viability assay. Def was tested in three different lung cell lines: A549, MRC5 CAF, and NL20 cells. Data showed the average of eight measurements (mean ± SD) of at least three independent experiments. (**B**) Early apoptosis activation by the phosphatidylserine (PS) externalization (green fluorescence). The upper images show the bright field. The NucBlue DAPI (blue) and Annexin V (green) fluorescence channels are overlaid in the bottom images. All images were taken at 40× magnification. Green fluorescence (525 nm) of Annexin V was statistically quantified ((**B**), down). Def was analyzed using confocal in duplicate in two independent experiments. The *p*-value thresholds for statistical significance were set as follows: **** for *p* < 0.0001.

To determine Def’s cytotoxicity, we began our analysis of Def determining its capability to eradicate malignant NSCLC, cancer-associated fibroblast (CAF) and normal cells. MTS viability assays were conducted on a NSCLC cell line, A549. Additionally, a CAF MRC5 cell line and normal bronchus NL20 cells were also treated with the Def compound for 24 h. Results shown in Figure 2A demonstrate that Def could kill both cancer cells and CAF in a dose-dependent manner. Def was more potent to A549, which had an IC_50_ of 95 µM, than to MRC5 (IC_50_ = 106 µM). However, the NL20 normal bronchus cells were affected in a similar cytotoxic pattern (IC_50_ = 97 µM) as NSCLC A549 cells. In Figure 2B, early apoptosis was studied via the externalization of phosphatidylserine (PS) on cell membrane using Annexin V (Alexa Fluor green dye) after a 24 h incubation with the IC_50_ (95 μM) of Def on A549 cells. Using confocal microscopy, it was determined that Def activated early apoptosis, as shown by the increased green dye intensity indicating PS externalization compared to untreated cells (*p* < 0.0001) (Figure 2B (down)). 

### 3.2. Def Promotes G0/G1 Cycle Arrest and DNA dsb

**Figure 3:**
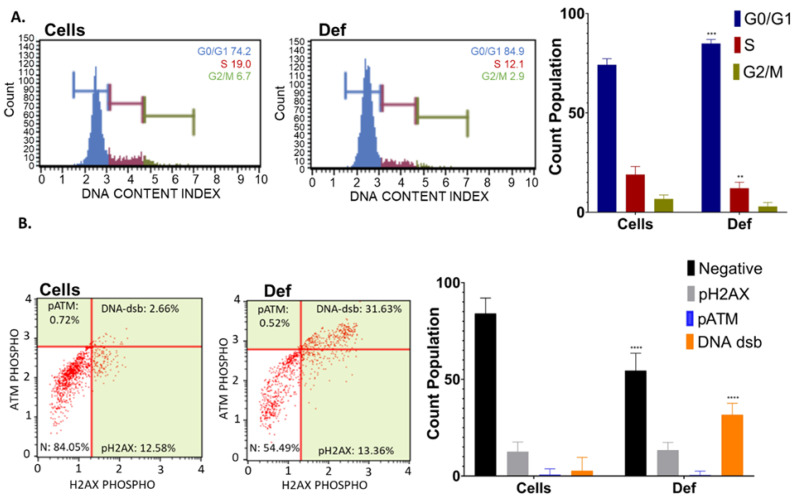
Nuclear-related mechanistic pathways. (**A**) Cell cycle arrest assay. Dot plot histograms (**left**) and bar graph with statistics (**right**). Def was analyzed in duplicate in two independent experiments. (**B**) DNA damage assay. Dot plot histograms (**left**) and bar graph with statistics (**right**). Def was analyzed in duplicate in two independent experiments The *p*-value thresholds for statistical significance were set as follows: **** for *p* < 0.0001, *** for *p* ranging from 0.0001 to 0.001 and ** for *p* between 0.01 and 0.001.

For nuclear-related mechanistic pathways, in Figure 3A, the population of cells present in each cell cycle phase was measured. Cells treated with Def showed a marked increase in the percentage of cells arrested in the G0/G1 phase and a decrease in cells in the S phase compared with untreated A549 cells. These results indicate that Def induces cell cycle arrest at the G0/G1 phase in NSCLC. Figure 3B presents a dot plot displaying distinct cellular states using antibodies to mark the DNA damage process: cells with no DNA damage, cells exclusively phosphorylated by ATM, cells solely phosphorylated by H2A.X, and cells featuring the co-activation of ATM and H2A.X, which is indicative of DNA double-strand breaks (dsb). After treatment with Def, 31.63% of cells showed an increase in DNA-dsb compared to 2.66% of untreated cells. The phosphorylation of histone and ATM alone remained unchanged despite Def treatment.

### 3.3. Def Induces Mitochondria Depolarization and Activation of Caspase 3/7

**Figure 4:**
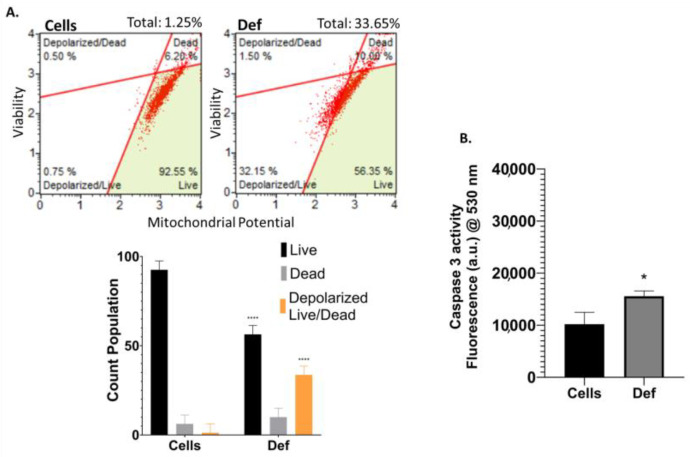
Mitochondrial-related mechanistic pathways. (**A**) Mitochondrial depolarization assay. This assay was performed in triplicates in two independent experiments. Dot plot histograms (**left**) and bar graph with statistics (**right**). (**B**) Caspase 3 activation. Data showed the average of four measurements (mean ± SD) of at least three independent experiments. The *p*-value thresholds for statistical significance were set as follows: **** for *p* < 0.0001, and * for *p* from 0.05 to 0.01.

The mitochondrion is inherently related to apoptosis. Thus, for mitochondrial-related mechanistic pathways, Figure 4A shows the effect of Def on mitochondrial membrane potential, a hallmark of intrinsic early apoptosis, and cellular plasma permeabilization, an indicator of late apoptosis. Def-treated cells demonstrated a significant increase in the mitochondrial depolarization of live and dead cells compared to untreated cells (*p* < 0.0001). Figure 4B shows the activity of caspase 3/7 after 24 h of Def treatment, indicating a significant activation of caspase 3 in treated cells (*p* < 0.05).

### 3.4. Def Chelates Iron Impairing Iron-Containing Proteins, GSH and Producing ROS

**Figure 5:**
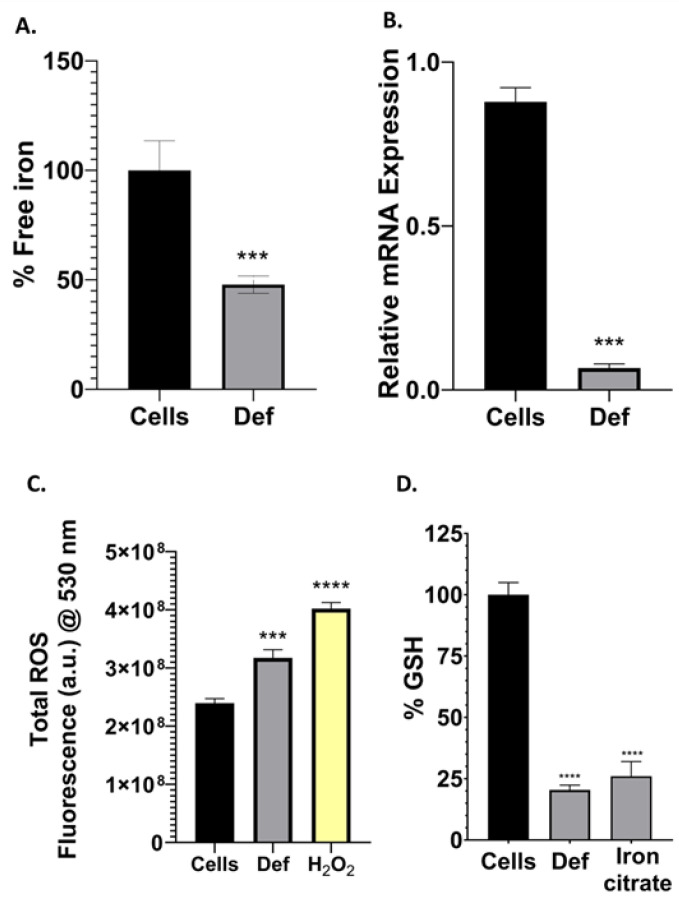
Iron-related processes. (**A**) Iron binding capacity assay. Treated and untreated cells were supplemented with a 200 μM iron citrate for this assay. Data showed the average of four measurements (mean ± SD) of at least three independent experiments. (**B**) RT-qPCR for *RR* mRNA gene expression. Data showed the average of three measurements (mean ± SD) of at least two independent experiments. (**C**) Total ROS production assay. H_2_O_2_ was used as a positive control. Data showed the average of four measurements (mean ± SD) of at least three independent experiments. (**D**) % GSH production. Iron citrate was used as a positive control. Data showed the average of four measurements (mean ± SD) of at least two independent experiments. The *p*-value thresholds for statistical significance were set as follows: **** for *p* < 0.0001 and *** for *p* ranging from 0.0001 to 0.001.

Iron is key for the overgrowth of cancer cells. Thus, to determine the intracellular iron binding capacity of Def, cells were harvested in a growth medium with iron citrate and after 24 h, were incubated with Def. Figure 5A shows that Def could bind iron and that there is substantially less free iron percentage after treatment with Def in A549 cells (*p* < 0.001). After we found iron impairment and mitochondrial damage due to Def, we decided to test if the expression of RR and the production of ROS were also affected. Figure 5B shows that Def induced the downregulation of the catalytic subunit *RRM1* gene compared to untreated cells (*p* < 0.001). Figure 5C exhibits the total ROS produced by the cells using the DCF dye. Def significantly induced the production of ROS (*p* < 0.001). Then, we decided to determine the effect of Def on the levels of cellular GSH, due to the high ROS concentration may decrease GSH. In Figure 5D, we elucidated Def capacity to decrease GSH levels.

### 3.5. Def Decreases Cell Migration, the Expression of ABCB1 Gene and EGFR Protein, and Upregulates NDRG1 Gene

**Figure 6:**
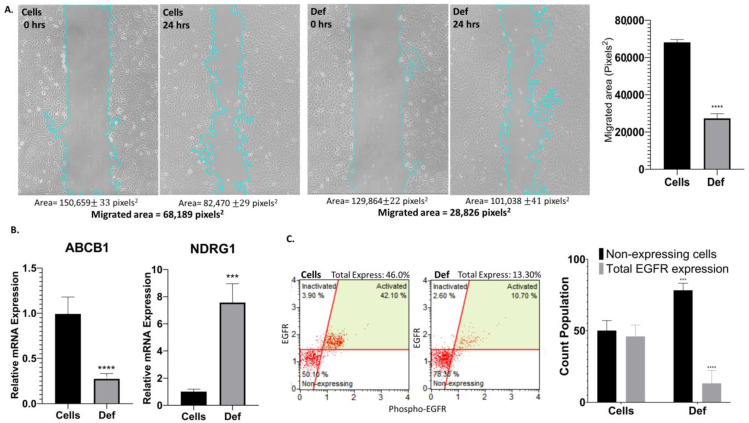
Migration and resistance-related processes. (**A**) Scratch Migration Assay (**left**) and statistics of the migration (**right**). Data showed the average of two measurements (mean ± SD) of at least three independent experiments. Blue lines show the measured area to determine migration. (**B**) mRNA Gene Expression of A549 cells treated with Def for 24 h using qPCR for *ABCB1* and *NDRG1* genes. Data showed the average of three measurements (mean ± SD) of at least two independent experiments. (**C**) EGFR expression and activation. Total Expression = Inactivated + Activated. Dot plot histograms (**left**) and bar graph with statistics (**right**). This assay was performed in triplicates in two independent experiments. The *p*-value thresholds for statistical significance were set as follows: **** for *p* < 0.0001 and *** for *p* ranging from 0.0001 to 0.001.

Invasion, metastasis, and chemoresistance are key processes in cancer that prevent its eradication. In Figure 6A, the migratory potential of the cells was assessed through a scratch wound healing assay. Def significantly decreased cell migration compared to untreated A549 cells (Figure 6A (right)) (*p* < 0.0001). From the genetic perspective, results after treatment with Def shown in Figure 6B demonstrated that *ABCB1*, a marker of chemoresistance, was downregulated (*p* < 0.0001), and *NDRG1*, a marker of metastasis suppression, was upregulated compared to untreated cells (*p* < 0.001). Then, we determined the expression of the EGFR protein after the exposure to Def. We found that after 24 h, Def significantly decreased the expression of the phosphorylated EGFR (*p* < 0.0001) in NSCLC cells (Figure 6C).

### 3.6. Def Is Safe and Diminishes Carcinoma Tumor Growth in Mice

**Figure 7:**
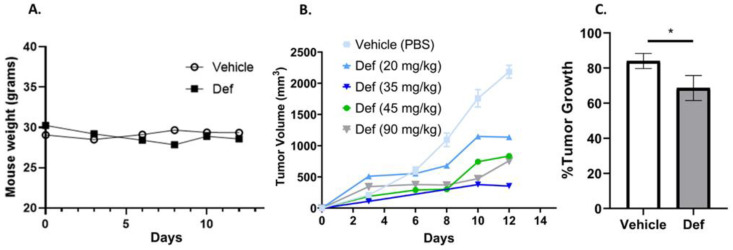
In vivo assays. (**A**) Average mouse weight of vehicle versus Def-treated mice was monitored on days 3, 6, 8, 10, and 12. (**B**) Tumor volume using a concentration range (20–90 mg/kg) of Def. (**C**) Tumor growth of vehicle vs. Def treated mice (from 20 to 90 mg/kg) after 12 days. Vehicle (n = 4) and Def-treated mice (n = 4). The *p*-value thresholds for statistical significance were set as * for *p* from 0.05 to 0.01.

Given the efficiency of Def decreasing the cell viability of lung carcinoma tumor cells, we decided to test its efficiency in reducing tumors in vivo using the LLC mouse model. Def was intraperitoneally administered from 20 to 90 mg/kg doses. First, we determined that the general health of our mice, proportional to their weight and grooming, were not affected during the Def treatment period. All the mice showed a stable weight (~30 g) during the 12 days (Figure 7A). Furthermore, in these 12 days, we observed a reduction in tumor volume in mice treated every 3 days with Def in all concentrations (Figure 7B). A calculation of the tumor growth percentage revealed 84% ± 8.6 tumor growth in vehicle-treated mice and 62% ± 8.7 in Def-treated mice (Figure 7C). The tumor growth of vehicle vs. Def (20, 35, 45, and 90 mg/kg) was performed manually using a caliper and results showed an 84% ± 8.6 tumor growth in vehicle-treated and 62% ± 8.7 in Def-treated mice (combined doses shown in column).

## 4. Discussion

This study explored the cytotoxic effects and potential mechanisms of Def in NSCLC cells, highlighting its potential role as a therapeutic agent in cancer treatment. While earlier research identified Def as a strong iron chelator [9,12,34], we specifically focused on how Def influences mechanistic pathways involving iron-regulated proteins in lung cancer cells.

The ability of cells to undergo apoptosis is critical for maintaining cellular homeostasis and eliminating malignant cells, with cancer cells often evading apoptosis through resistance mechanisms [35]. Given this, it is important to understand how Def influences cytotoxicity and apoptotic pathways in cancer cells. MRC5 cells, despite being normal fibroblasts, contribute to cancer cell proliferation and cancer stem cell marker expression [36]. We demonstrated that Def could induce cell death in both NSCLC A549 cells and CAF MRC5 cells in a dose-dependent manner, showing greater toxicity toward A549 cells compared to MRC5 cells. Notably, the NL20 normal bronchus cells exhibited a similar cytotoxic response to A549 cells. Basically, Def did not show selective cytotoxicity in vitro. Def also promoted early apoptosis in NSCLC A549 cells, as indicated by the externalization of PS.

Cell cycle regulation plays a vital role in developing more effective cancer treatments, particularly in understanding how specific compounds may impact various cell cycle phases. Following treatment with Def, a substantial increase in the proportion of cells in the G0/G1 phase was observed, accompanied by a decrease in cells in the S phase and G2/M phase. These observations imply that Def induces cell cycle arrest at the G0/G1 phase. Similar findings were reported in studies involving leukemia cells [37] and hepatocytes [38]. This aligns with the notion that iron deprivation can primarily cause cell cycle arrest at the G1/S transition [39].

DNA damage is a key target in chemotherapy to kill cancer cells. We demonstrated that Def increased DNA-dsb by the phosphorylation of ATM and H2A.X. This DNA damage could potentially account for the reduced S phase observed, as DNA disruption may trigger cell cycle arrest at the checkpoint preceding the S phase [40]. Previous work by Samara et al., 2017 indicated that Def induced severe DNA damage in mantle cell lymphoma cells [41].

Intrinsic apoptosis is a programmed cell death mechanism that plays a crucial role in response to a variety of cellular stresses, including DNA damage [42]. The loss of mitochondrial membrane potential is an early sign of apoptosis initiation and an increase in cellular plasma permeabilization is indicative of late apoptosis [42,43]. We elucidated that Def showed significant mitochondrial depolarization, alongside an increased population of dead cells. Earlier studies reported that Def induces mitochondrial potential loss [44]. These observations confirm that Def triggers intrinsic apoptosis, likely due to its impact on the dysregulation of intracellular iron homeostasis and the induction of DNA dsb.

Upon mitochondrial depolarization, apoptosis proceeds through caspase 3 activation, facilitated by the release of cytochrome c from mitochondria [45]. We found that Def significantly increases the activation of the caspase 3/7 enzyme. Despite this increase, caspase 3 activity was not extremely high, potentially due to Def’s chelating properties reducing the activity of the electron transport chain (ETC) complexes and cytochrome c iron content. Caspase 3 activity is contingent upon the formation of the apoptosome complex, comprising cytochrome c and Apaf-1 [46].

Given Def’s role as an iron chelator and considering that one strategy in cancer therapy involves disrupting iron metabolism, evaluating the levels of free iron and the effect on iron-containing proteins in NSCLC cells is essential. A marked reduction in free iron levels post-treatment with Def suggests its efficacy as an intracellular iron chelator. Consistent with these observations, prior studies showed that Def binds free iron in normal kidney and esophageal cancer cells [47,48]. Additionally, Def was shown to form stable anti-cancer compounds with titanium, which implies its chelation capabilities may extend beyond iron [49]. Thus, the impact of Def on RR is very important because it is an iron-regulated protein [16]. Also, RR has a key role catalyzing the rate-limiting step in DNA synthesis [19] and we found that Def arrested NSCLC cells in G0/G1 step. We demonstrated that Def reduced the *RRM1* mRNA expression in NSCLC A549 cells. Research demonstrated that iron chelators can disrupt the iron supply required for the beta subunit of RR, resulting in the inactivation of RRM1 and RRM2 [16]. Moreover, enzymatic assays showed that Def can inhibit RR activity [41]. This downregulation may also be linked to the observed reduction in free iron levels due to Def’s chelation effect, considering the iron dependency of RR function.

ROS play a crucial role in various cellular processes, including hypoxia, apoptosis, and proliferation [50]. Mitochondria are the primary source of cellular ROS, particularly those produced at the ETC [51]. Def demonstrated significantly increased ROS production identified by the conversion of O_2_, (the final electron acceptor of the ETC) to H_2_O_2_. The increase in ROS is likely due to the iron depletion of ETC complexes, resulting in the release of electrons to react with oxygen. Interestingly, we observed that this increase in ROS also led to a reduction in intracellular GSH levels. While other iron chelators, such as deferoxamine and deferiprone, were shown to prevent ferroptosis by elevating GSH levels [52], the oxidative stress and GSH depletion caused by Def may induce ferroptosis in NSCLC cells. In the literature, we found a recent article studying the effect of Def on leukemia cells that confirms the potential of Def to induce ferroptosis [53].

Cancer cell invasion and metastasis, key contributors to cancer-related mortality, represent significant therapeutic challenges [54]. The migratory potential of A549 cells was evaluated using a scratch wound healing assay. Def substantially reduced cell migration, indicative of its anti-invasion potential. Previous research indicated that reduced cellular iron levels can suppress migration and invasion [28]. Chemoresistance is also a challenge in cancer therapy, often resulting in poor patient outcomes and limiting the effectiveness of treatments [55,56]. Thus, *ABCB1*, a key player in drug resistance, and *NDRG1*, a metastasis suppressor in various cancers, were studied as main targets to improve therapy outcomes [21,24]. We demonstrated that Def promotes the downregulation of *ABCB1* and upregulation of *NDRG1* in NSCLC A549 cells. Research indicated that *NDRG1* expression is modulated by cellular iron depletion, with iron chelators capable of enhancing *NDRG1* gene expression [57,58]. Def was previously reported to increase NDRG1 levels in cervical, prostate, and colon cancer cells [29,58]. These findings further support the notion that Def disrupts iron homeostasis through its chelating effect. Another critical hallmark is the aberrant activation of EGFR because it is associated with increased cellular proliferation, angiogenesis, metastasis, and chemoresistance in NSCLC therapies [59]. Def treatment markedly decreased EGFR expression and phosphorylation. This reduction in EGFR expression aligns with the observed downregulation of *ABCB1* and upregulation of *NDRG1*, reinforcing the therapeutic potential of Def in targeting EGFR-driven oncogenic pathways. Thus, Def appears to effectively disrupt processes central to cancer progression, such as metastasis and chemoresistance, potentially through its multifaceted impact on EGFR, *ABCB1*, and NDRG1 pathways, in support of our initial hypothesis. Recent studies also demonstrated that the iron chelator deferiprone can reduce metastasis in ovarian cancer [60].

Following the assessment of Def’s effects on various cellular processes, Def safety and efficacy in vivo is critical to demonstrate its clinical potential. While significant weight loss (greater than 20% loss during treatment) could suggest adverse effects like poor appetite or systemic toxicity, weight gain might indicate conditions such as fluid retention or metabolic imbalances in tumor models [61]. The stable and comparable weight outcomes between Def treatments suggest that it does not adversely impact the overall health of the mice. In addition, our syngeneic model, with an active immune system, also offers a more realistic scenario for predicting Def’s performance against NSCLC, considering the immune system’s role in tumor development. Previous studies demonstrated the anti-tumor effects of Def in mice when administered orally [29,48,62]. To provide initial insights into the dose-dependent efficacy of Def via subcutaneous injection, the present study was conducted on a small group of mice. The findings could indicate a tendency for a reduction in tumor volume with increasing doses of Def and a statistically significant reduction in tumor growth when all concentrations were grouped together and compared to the vehicle. While these results are promising, we acknowledge the limitations of our study due to the small sample size. To definitively validate the anti-cancer efficacy of Def and ensure reproducibility, future studies with larger sample sizes per treatment group are warranted.

## 5. Conclusions

This study aimed to identify how the iron chelator Def impacts NSCLC and its effect on key cellular mechanistic pathways, especially against chemoresistance and metastasis. We demonstrated that Def reduced cell viability and induced early apoptosis in NSCLC cells. However, Def did not show great selectivity over NSCLC to normal lung cells. On the other hand, to resolve this problem, future experiments could be focused on Def encapsulation into targeted drug delivery systems to increase its specificity. Def also reduced free iron in cancer cells, as demonstrated in clinical use. Furthermore, Def arrested NSCLC cells in the G0/G1 cell cycle phase by promoting DNA-dsb and the downregulation of the catalytic subunit of the RR complex. Def induced intrinsic apoptosis by mitochondrial depolarization and caspase 3/7 activation. Def also induced a high production of ROS that decreased GSH levels. In addition, Def demonstrated that it could counteract three of the main cancer resistance and metastasis-mediated mechanisms. In this way, EGFR and *ABCB1* were downregulated, and *NDRG1* was upregulated by Def, highlighting the possibilities of Def overcoming drug resistance and invasion. In vivo experiments in a syngeneic mouse model showed that Def was non-toxic at the tested concentrations and suggests a decrease in the tumor volume. Future directions of our project include exploring Def’s properties in combination with current chemotherapies (e.g., cisplatin and doxorubicin) for NSCLC to determine if Def can work in synergy with chemotherapy to increase its effectiveness and diminish secondary side effects.

## Data Availability

All data and materials used are available from the corresponding author upon reasonable request.
